# A case report: unmasking a singular culprit for cardiogenic shock: looking beyond the coronary tree

**DOI:** 10.1093/ehjcr/ytz009

**Published:** 2019-03-28

**Authors:** Luís Almeida-Morais, Guilherme Portugal, Rui Cruz-Ferreira

**Affiliations:** Centro Universitário de Cardiologia, Hospital de Santa Marta, Centro Hospitalar de Lisboa, Rua de Santa Marta, n° 50, Lisbon, Portugal

**Keywords:** Cardiogenic shock, Lung cancer, Multimodality imaging, Case report

## Abstract

**Background:**

Cardiogenic shock remains challenging in its therapy and aetiology.

**Case summary:**

A 74-year-old woman admitted for cardiogenic shock requiring mechanical ventilation and high-dose inotropics and vasopressors with an electrocardiogram showing left ventricular (LV) lateral wall ischaemia had diffuse coronary artery disease but TIMI III flow in the coronary tree. An echocardiogram showed a suspicious mass invading the left ventricle and computed tomography scan revealed an advanced lung cancer with LV wall and pulmonary artery invasion as the cardiogenic shock cause.

**Discussion:**

When managing cardiogenic shock, it is important to consider different and not obvious diagnosis. A high level of clinical suspicion and multimodality imaging assessment was very important in the present case to attain the diagnosis.

**Learning points**

- Cardiogenic shock still represents a challenge condition to diagnose and managing.
- Multimodality fast access imaging should be used in the non-typical cases of cardiogenic shock to clarify their aetiology.
- A high level of clinical suspicion is needed for some challenging cases of cardiogenic shock.

## Introduction

Cardiogenic shock is defined by a decrease in cardiac output and evidence of tissue hypoxia despite adequate intravascular volume.^1^ The reduced cardiac output induces compensatory mechanisms that are usually not satisfactory and if no interventions are undertaken, multi-organ failure and death occur.^2^ In the contemporary era, cardiogenic shock patients’ management remains challenging. The most frequent cause for this condition is still ischaemic heart disease with a prevalence of 6–10% following ST-elevation myocardial infarction.^3^ Moreover, despite all advances in medical therapy, percutaneous coronary intervention and left ventricular (LV) assist devices, in-hospital mortality rate remains high.^4^

## Timeline

**Table:** 

Investigations	Findings
Clinical presentation	Acute dyspnoea and haemodynamic collapse
Electrocardiogram	Right bundle branch block and lateral ST elevation
Coronary angiogram	No coronary artery occlusion
Echocardiogram	Left ventricular dysfunction and kinetic changes
Computed tomography scan	No pulmonary embolism. Left ventricle and pulmonary artery invading tumour

## Case presentation

**Figure 1:**
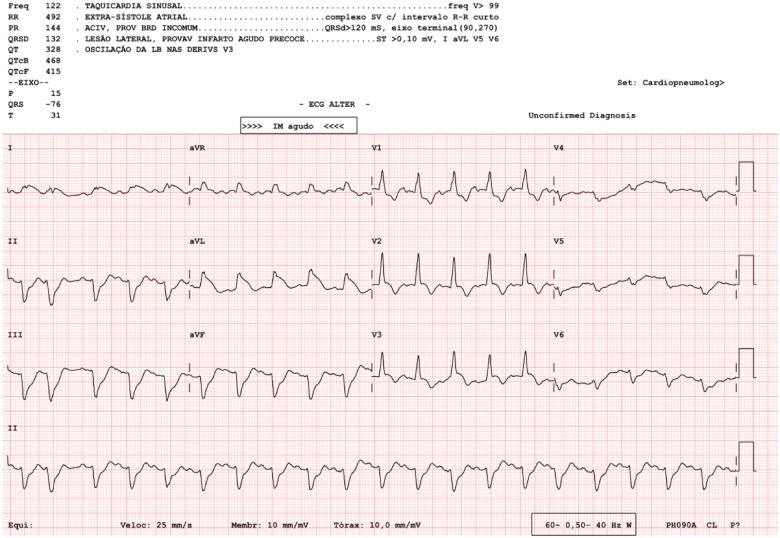
Patient’s 12-lead electrocardiogram at presentation shows sinus tachycardia, right bundle brunch block, and left anterior hemiblock with 2 mm ST-elevation in leads aVL, aVR, and I.

**Figure 2:**
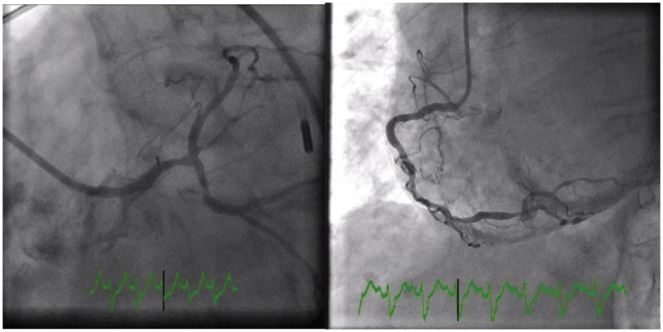
Coronary angiography shows three vessel diffuse disease with TIMI flow III in all vessels.

**Figure 3:**
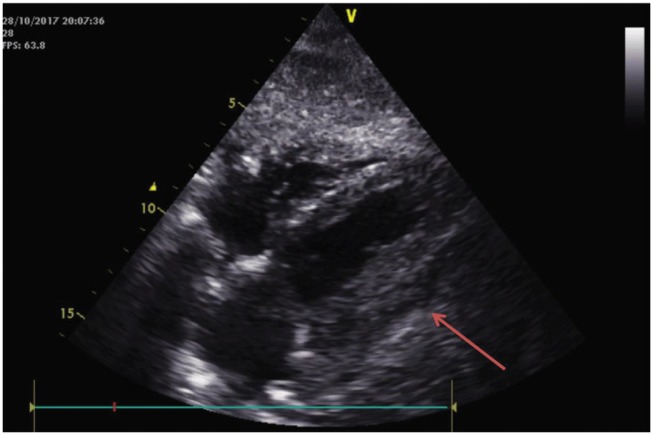
Transthoracic echocardiography four-chamber subcostal view showing a echogenic mass adjacent to the lateral left ventricular wall.

**Figure 4:**
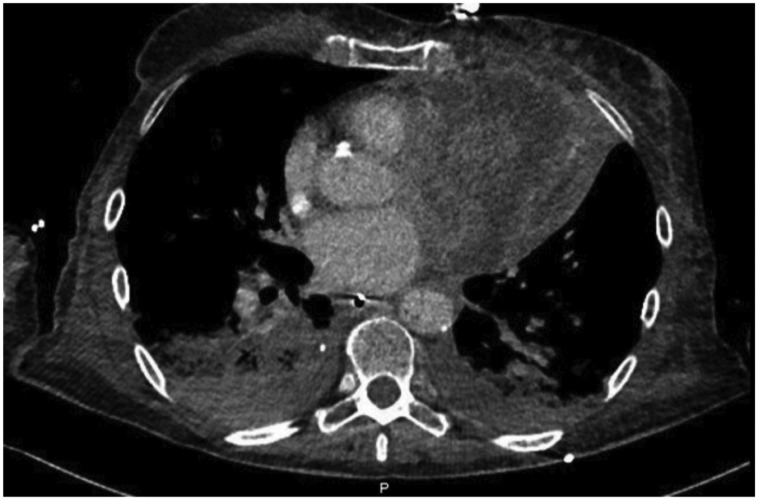
Thoracic computed tomography scan showing a heterogeneous mass, with necrotic material, originating from the inferior left lung lobe invading the anterior and lateral wall of the left ventricle, left branch of the pulmonary artery, left bronchi and anterior chest wall.

A 74-year-old woman with past medical history of left breast cancer submitted to radical mastectomy 10 years ago was admitted to the emergency department for acute dyspnoea. Clinical observation showed tachycardia, blood pressure 89/54 mmHg, regular heart sounds, no heart murmurs, arterial oxygen saturation of 85%, tachypnoea, accessory respiratory muscles use, and bilateral rales with left hemithorax dullness at percussion. Electrocardiogram showed sinus tachycardia, right bundle brunch block, and left anterior hemiblock with 2 mm ST-elevation in leads aVL, aVR, and I (*Figure 1*). Clinical condition deteriorated requiring intensive care unit admission, inotropic and vasopressor support, and mechanical ventilation. Bedside transthoracic echocardiogram (TTE) showed impaired LV function with anterior and lateral wall akinesia. A presumptive diagnosis of myocardial infarction (MI) presenting as cardiogenic shock was made and antithrombotic therapy including loading doses of aspirin (250 mg), ticagrelor (180 mg), and heparin (5000 UI) were administered. Emergent coronary angiogram was performed and showed diffuse non-significative three-vessels disease (*Figure 2*). Cardiac biomarkers were elevated (high sensitivity troponin I 32 ng/mL, for a normal <0.07 ng/mL and BNP 528 pg/mL, for a normal <100 pg/mL). Based on radiological chest imaging, a mass in the left lung was suspected; TTE imaging review showed LV lateral and anterior wall akinesis due to infiltration by a heterogeneous echogenic mass (*Figure 3*). Transoesophageal echocardiogram confirmed TTE findings, showing mild mitral regurgitation and a large mass invading the lateral LV wall. An urgent computed tomography (CT) scan was performed showing a neoformation located in the lower and mid sections of the left hemithorax, invading the lateral LV wall, pulmonary artery left branch, left pulmonary bronchi and anterior thoracic wall, compatible with an advanced lung cancer (*Figure 4*). Despite supportive care, clinical status worsened and the patient died in the following hours.

## Discussion

To the authors; knowledge, this is the first report of lung cancer mimicking a MI with cardiogenic shock. Despite the absence of typical chest pain, the present case was at first assessed as a cardiogenic shock complicating a MI. The electrocardiogram and echocardiogram findings, and later the cardiac enzymes, suggested left ventricle ongoing ischaemia. However, the coronary angiography revealed no coronary artery occlusion or significant stenosis explaining the clinical presentation. Coronary arteries presented diffuse disease with small vessels and a rich collateral circulation. Revision of the echocardiography images revealed a suspicious mass attached to the left ventricle, what immediately conducted to further imaging modalities. Both transoeosophageal echocardiogram and CT scan were helpful to the diagnosis and haemodynamic impact of a tumoural mass invading the LV wall and pulmonary artery. We believe that the cardiogenic shock mechanism in this case is explained in part by the dysfunction caused by the LV anterior and lateral akinesia with LV dysfunction, but also by the pulmonary artery obstruction which impairs adequate pre-load to the left ventricle. Moreover, the pro-inflammatory and high metabolic rate status that characterizes an advanced and invasive tumour might also contribute for the haemodynamic collapse.

## Supplementary material

Supplementary material is available at *European Heart Journal - Case Reports* online.

**Slide sets:** A fully edited slide set detailing this case and suitable for local presentation is available online as Supplementary data.

**Consent:** The author/s confirm that written consent for submission and publication of this case report including image(s) and associated text has been obtained from the patient in line with COPE guidance.

**Conflict of interest:** none declared.

## Supplementary Material

ytz009_Slide_Set
