# Supplementary material for: A case report: unmasking a singular culprit for cardiogenic shock: looking beyond the coronary tree
Source: Eur Heart J Case Rep. 2019 Mar 28;3(2):ytz009. doi: 10.1093/ehjcr/ytz009 (PMC6601217; doi:10.1093/ehjcr/ytz009)
Supplement: ytz009_Slide_Set [file ytz009_slide_set.pptx]

## Slide 1
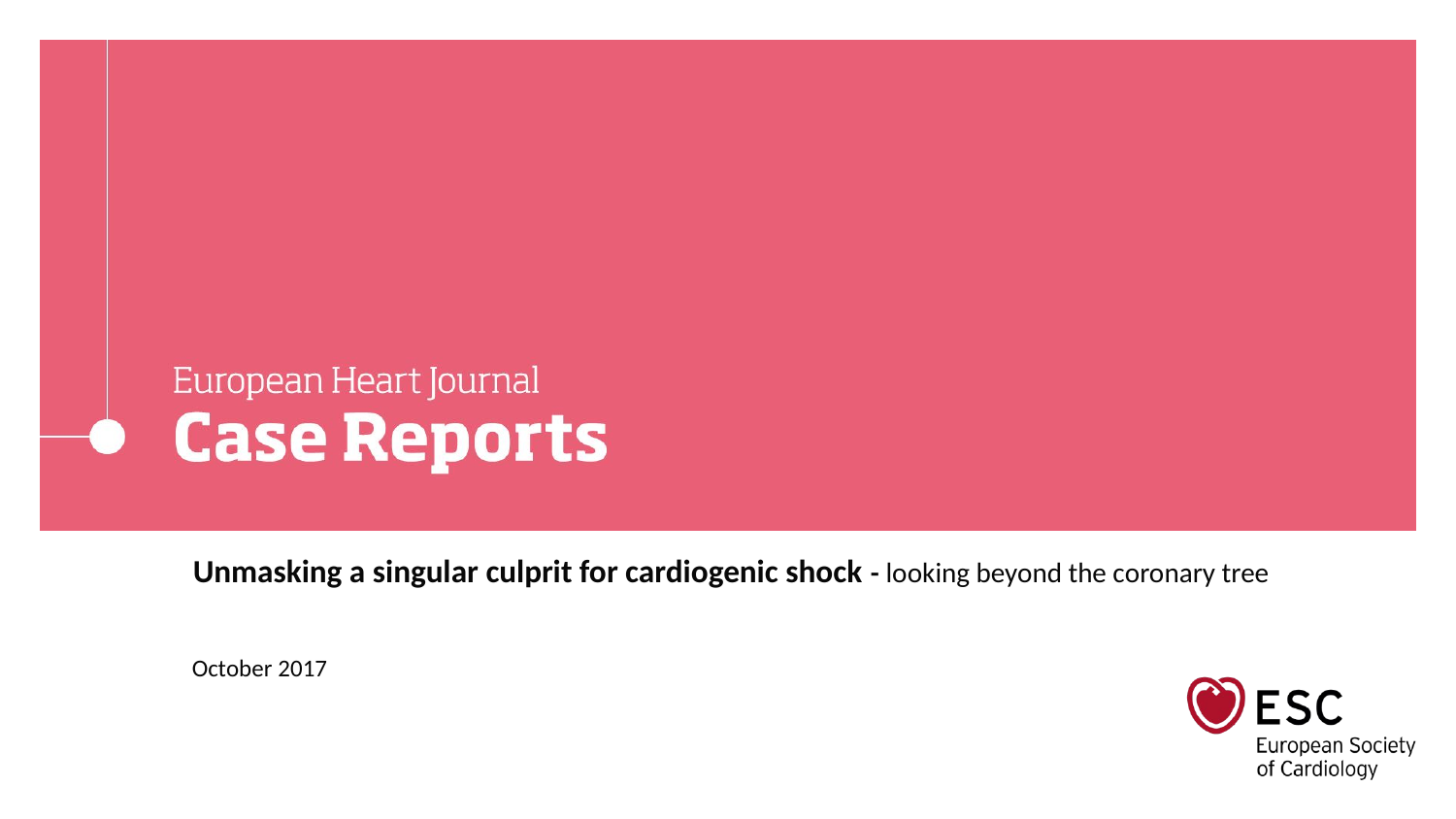

# Unmasking a singular culprit for cardiogenic shock - looking beyond the coronary tree
October 2017

## Slide 2
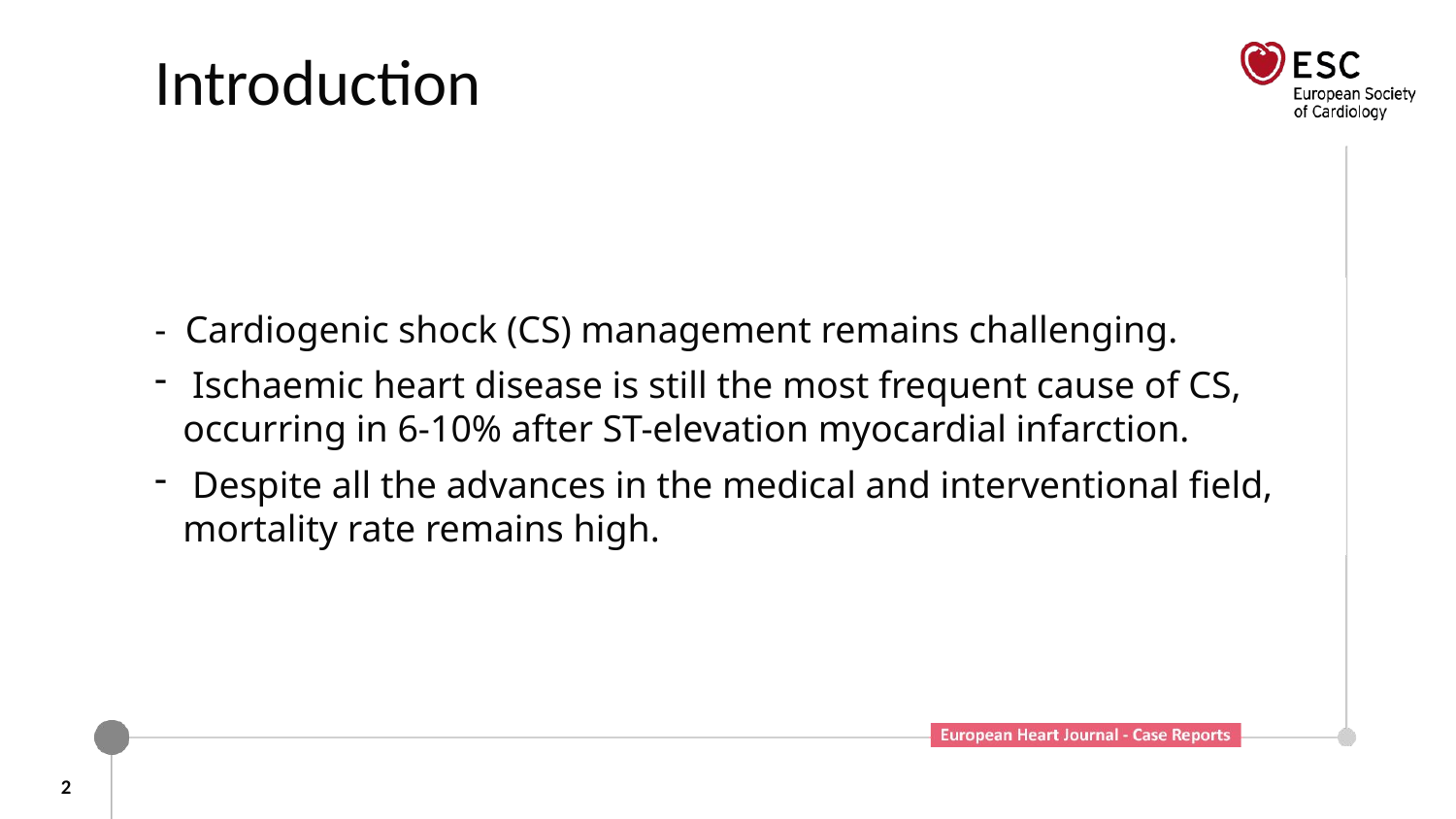

# Introduction
- Cardiogenic shock (CS) management remains challenging.
 Ischaemic heart disease is still the most frequent cause of CS, occurring in 6-10% after ST-elevation myocardial infarction.
 Despite all the advances in the medical and interventional field, mortality rate remains high.
2

## Slide 3
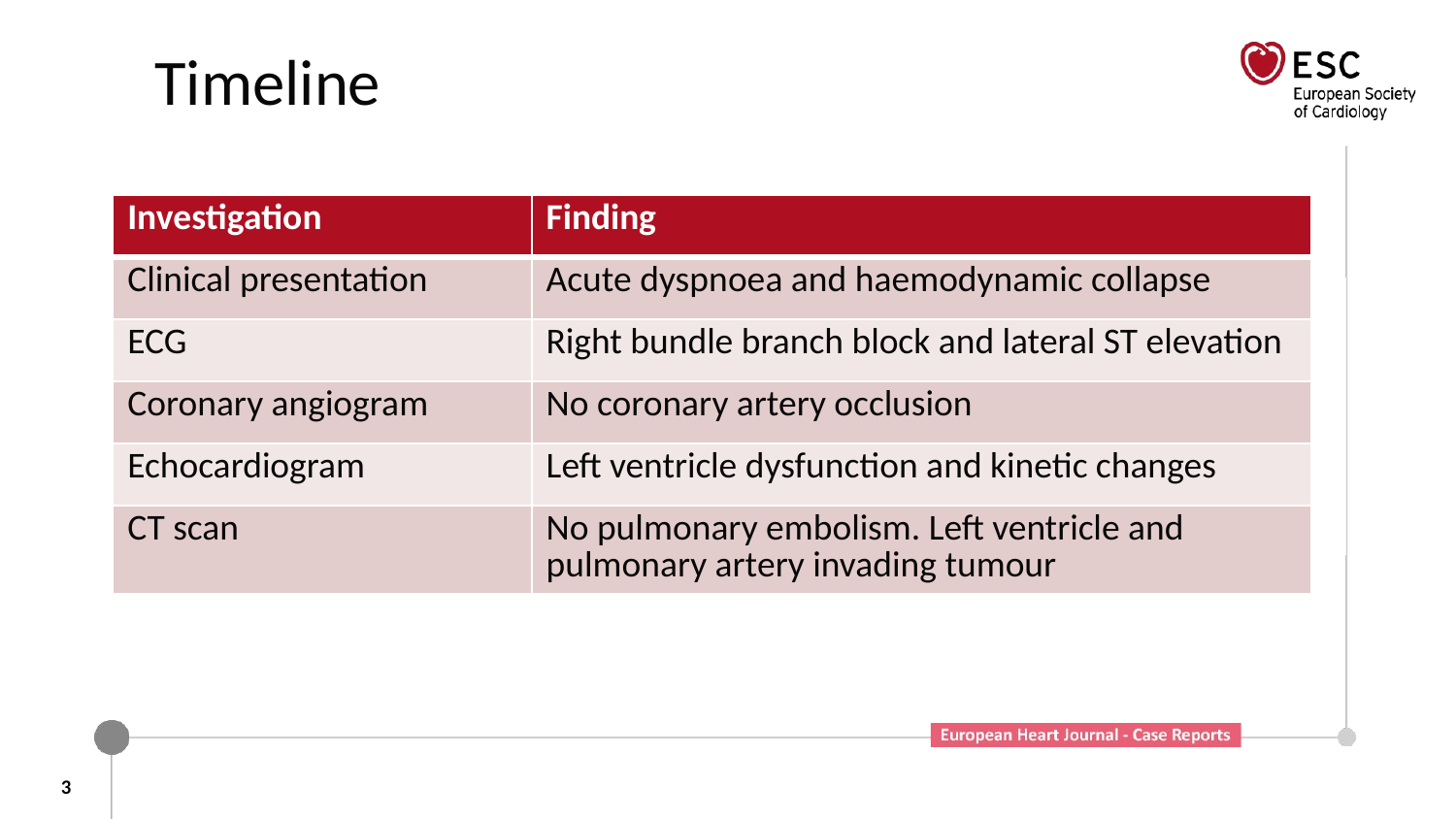

# Timeline
| Investigation | Finding |
| --- | --- |
| Clinical presentation | Acute dyspnoea and haemodynamic collapse |
| ECG | Right bundle branch block and lateral ST elevation |
| Coronary angiogram | No coronary artery occlusion |
| Echocardiogram | Left ventricle dysfunction and kinetic changes |
| CT scan | No pulmonary embolism. Left ventricle and pulmonary artery invading tumour |
3

## Slide 4
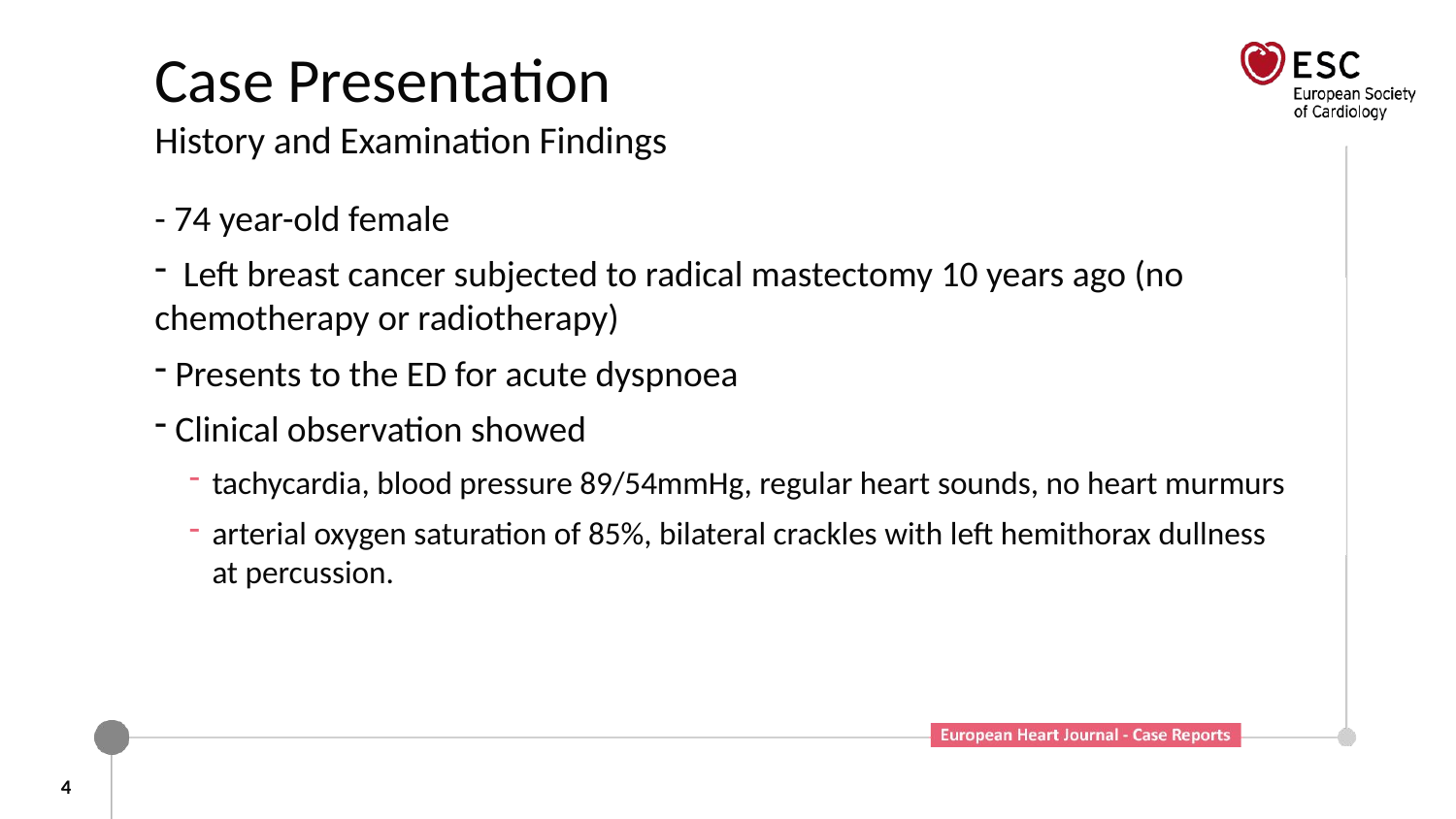

# Case Presentation History and Examination Findings
- 74 year-old female
 Left breast cancer subjected to radical mastectomy 10 years ago (no chemotherapy or radiotherapy)
 Presents to the ED for acute dyspnoea
 Clinical observation showed
tachycardia, blood pressure 89/54mmHg, regular heart sounds, no heart murmurs
arterial oxygen saturation of 85%, bilateral crackles with left hemithorax dullness at percussion.
4

## Slide 5
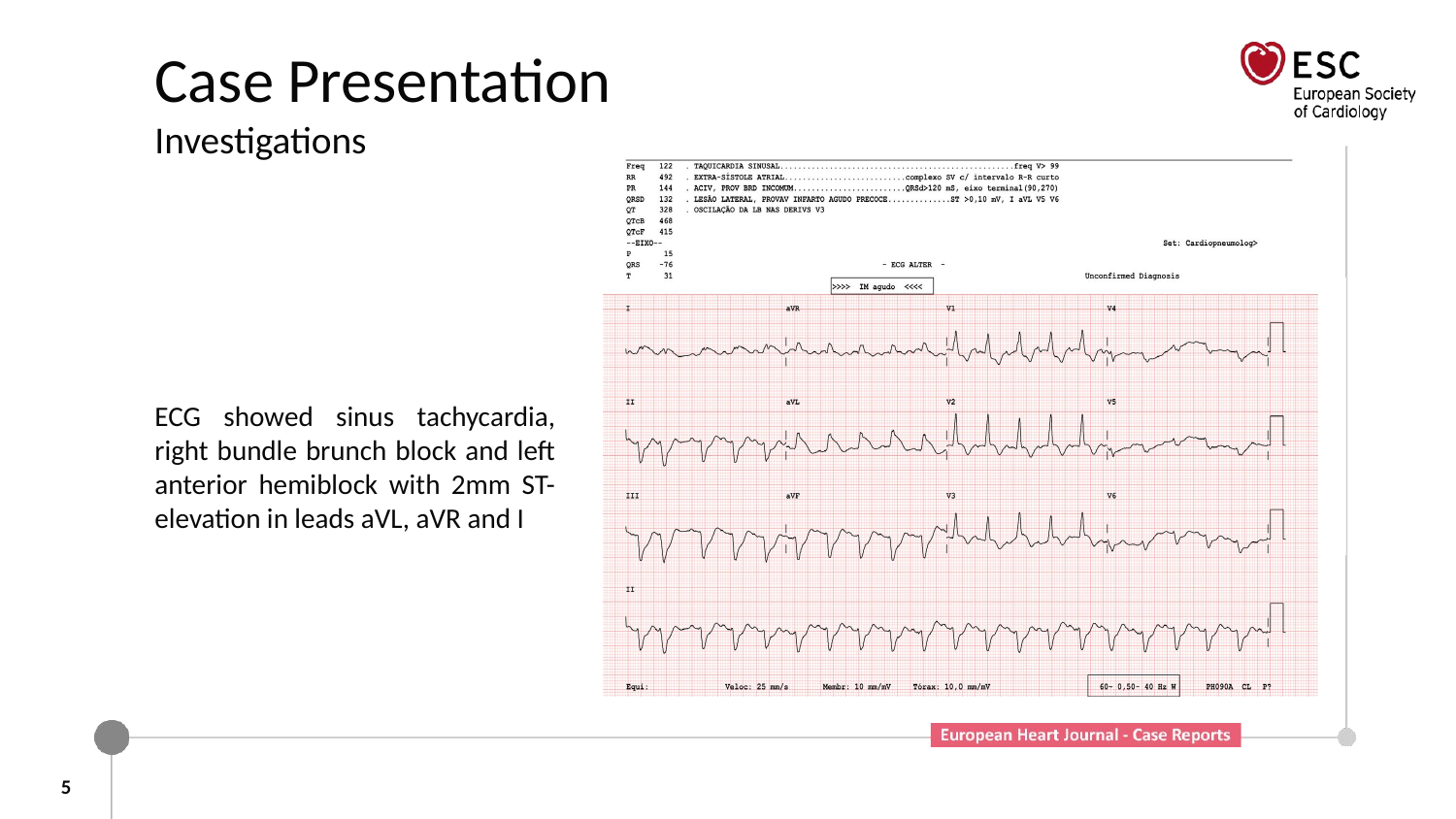

# Case Presentation Investigations
ECG showed sinus tachycardia, right bundle brunch block and left anterior hemiblock with 2mm ST-elevation in leads aVL, aVR and I
5

## Slide 6
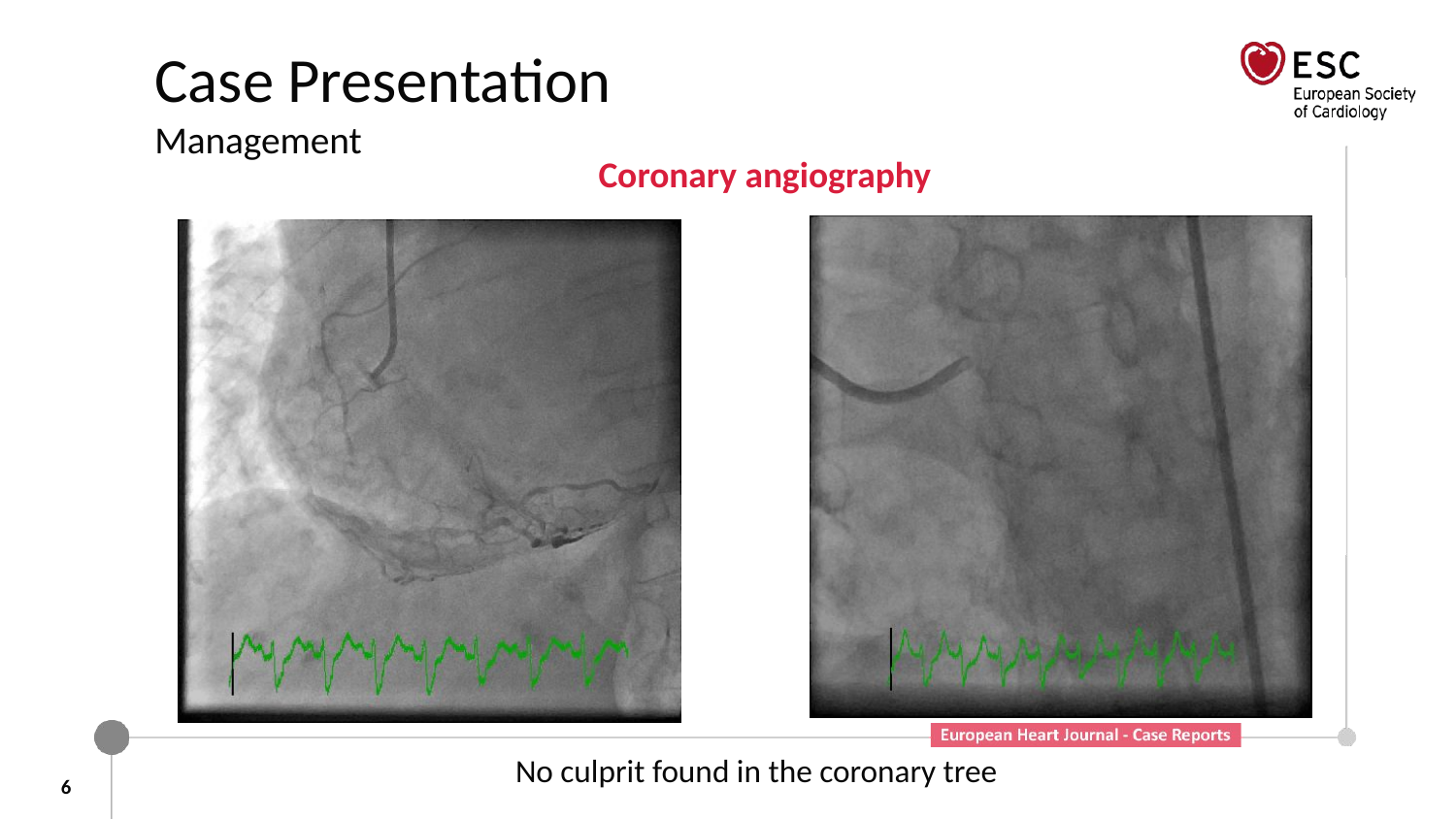

# Case Presentation Management
Coronary angiography
No culprit found in the coronary tree
6

## Slide 7
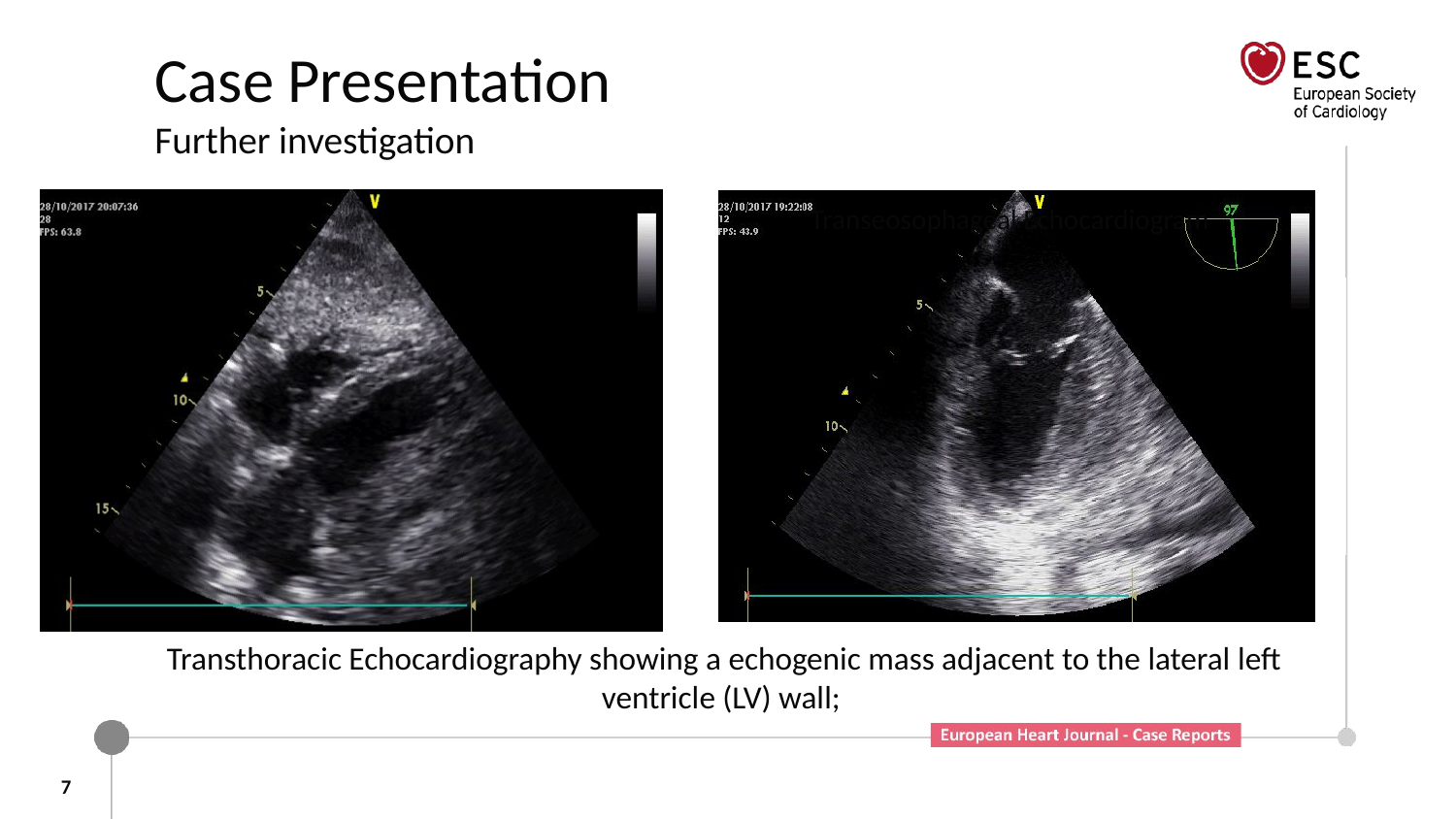

# Case Presentation Further investigation
Transeosophageal Echocardiogram
Transthoracic Echocardiography showing a echogenic mass adjacent to the lateral left ventricle (LV) wall;
7

## Slide 8
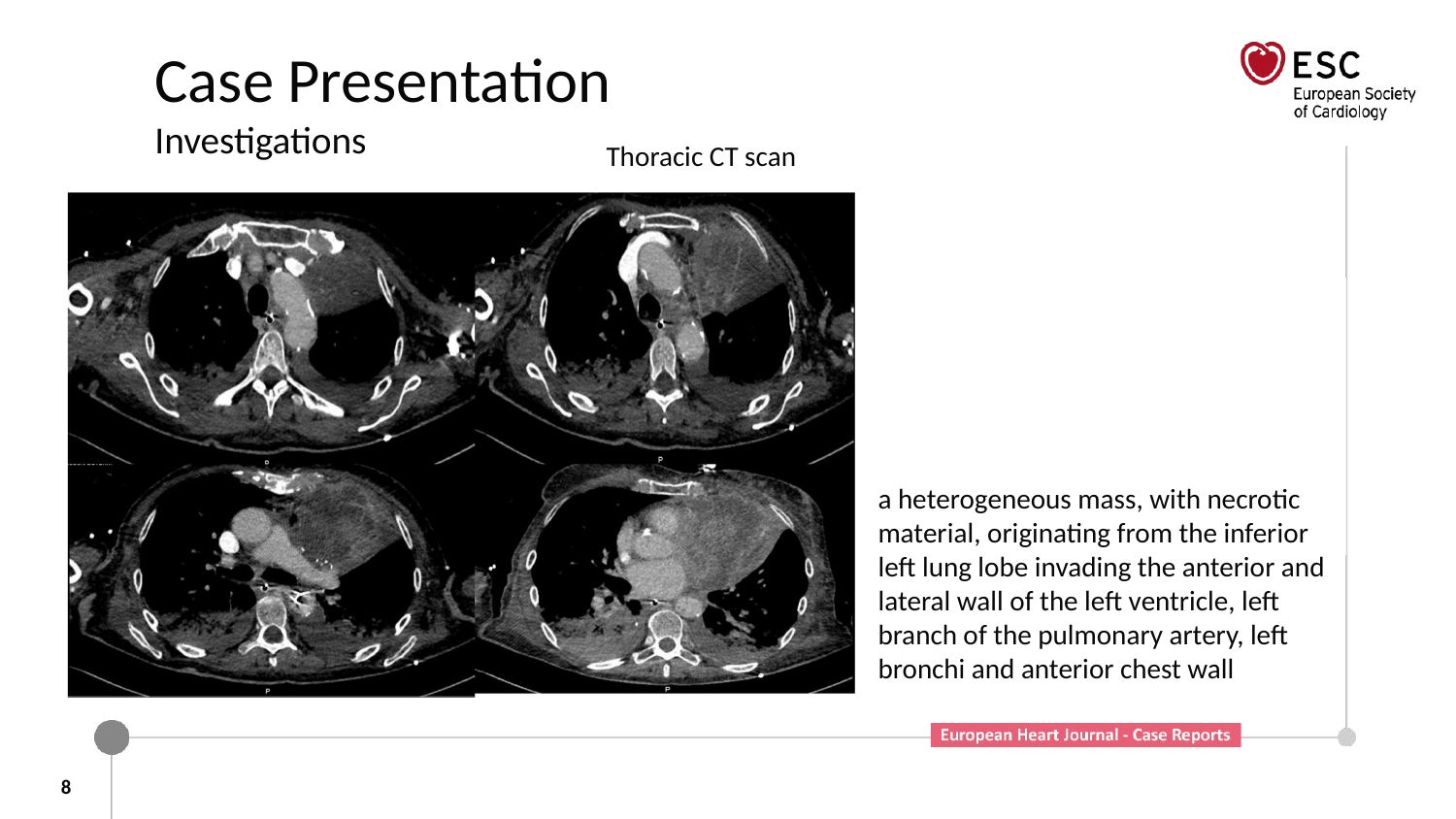

# Case Presentation Investigations
Thoracic CT scan
a heterogeneous mass, with necrotic material, originating from the inferior left lung lobe invading the anterior and lateral wall of the left ventricle, left branch of the pulmonary artery, left bronchi and anterior chest wall
8

## Slide 9
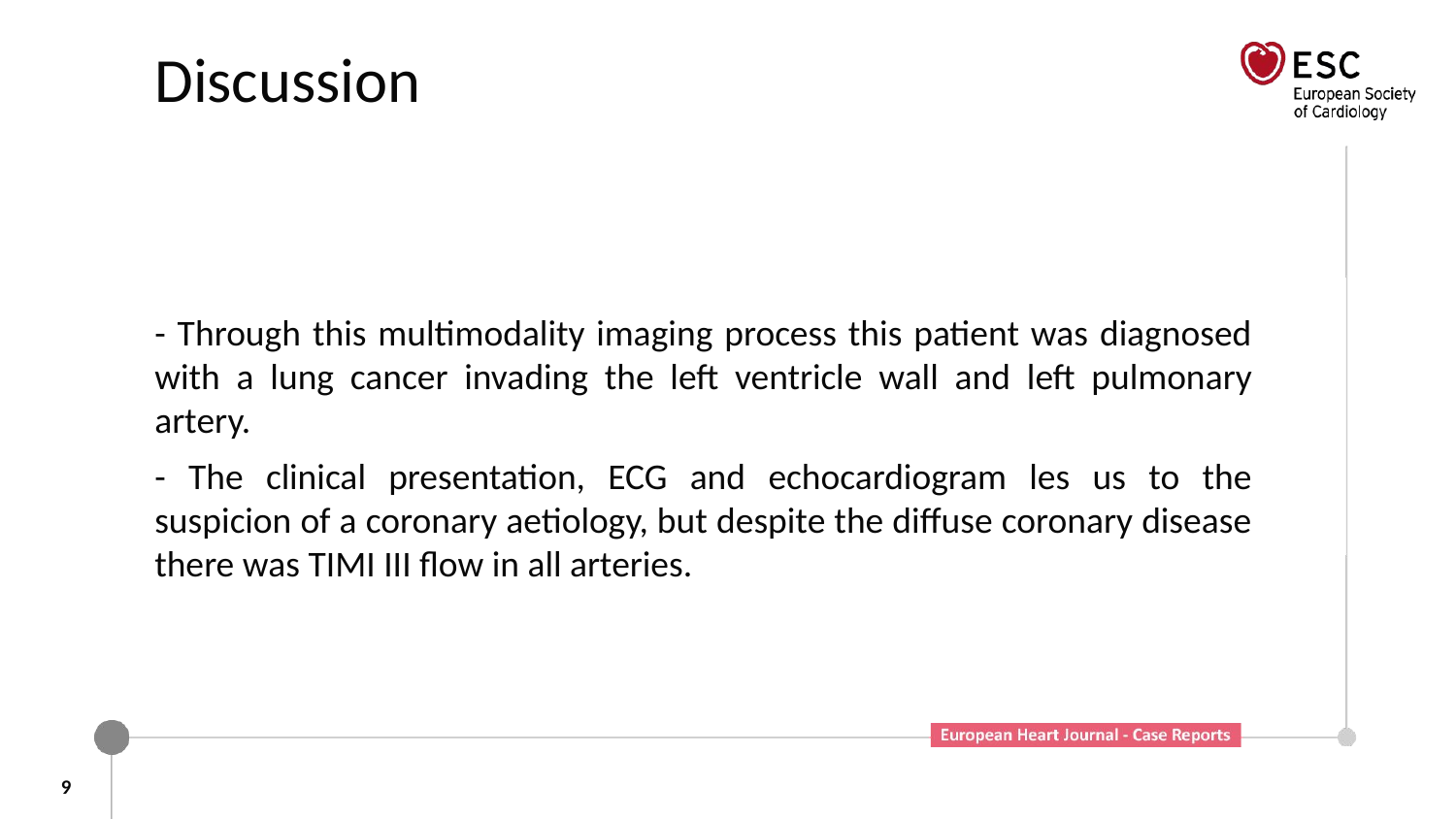

# Discussion
- Through this multimodality imaging process this patient was diagnosed with a lung cancer invading the left ventricle wall and left pulmonary artery.
- The clinical presentation, ECG and echocardiogram les us to the suspicion of a coronary aetiology, but despite the diffuse coronary disease there was TIMI III flow in all arteries.
9

## Slide 10
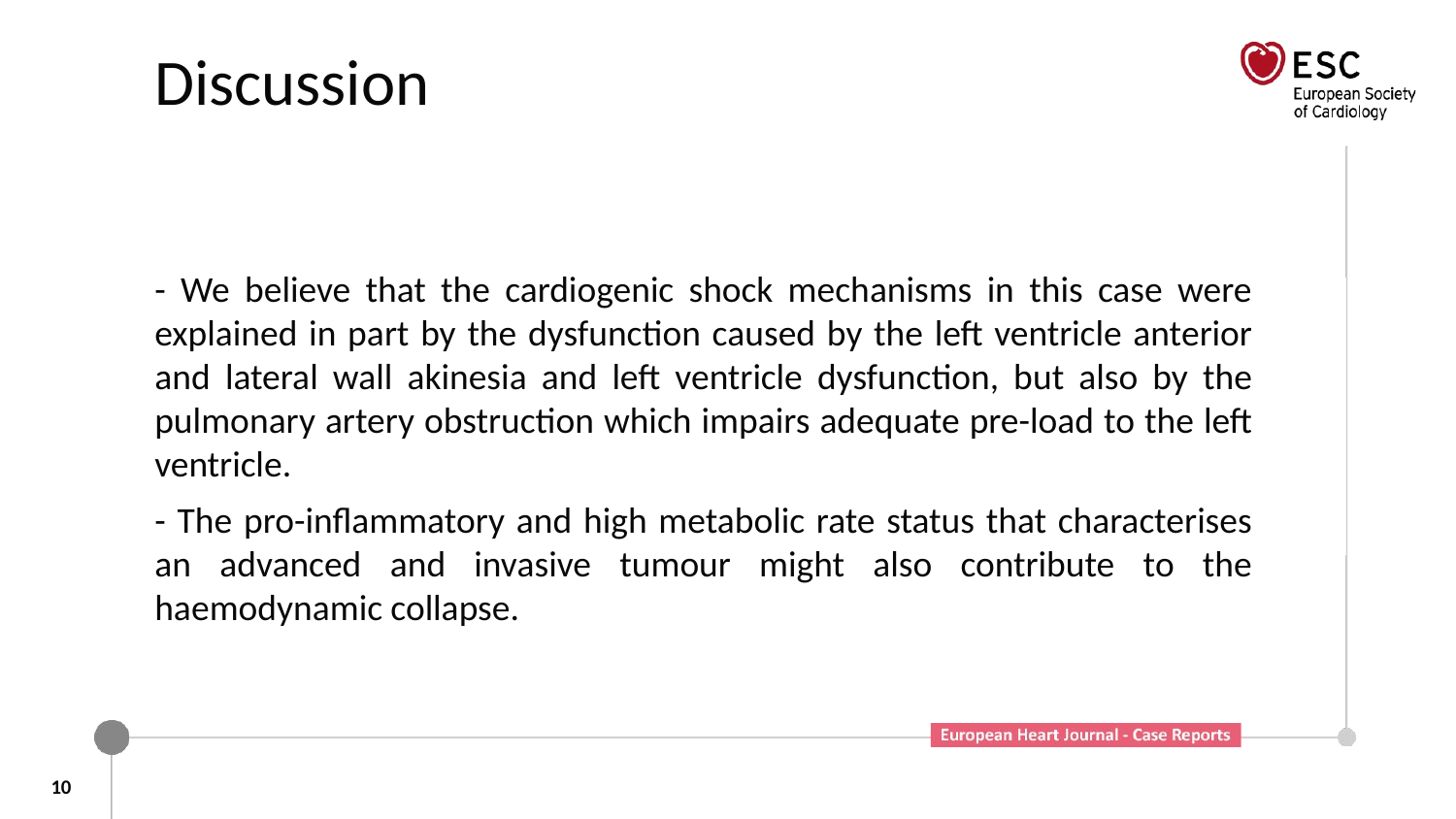

# Discussion
- We believe that the cardiogenic shock mechanisms in this case were explained in part by the dysfunction caused by the left ventricle anterior and lateral wall akinesia and left ventricle dysfunction, but also by the pulmonary artery obstruction which impairs adequate pre-load to the left ventricle.
- The pro-inflammatory and high metabolic rate status that characterises an advanced and invasive tumour might also contribute to the haemodynamic collapse.
10

## Slide 11
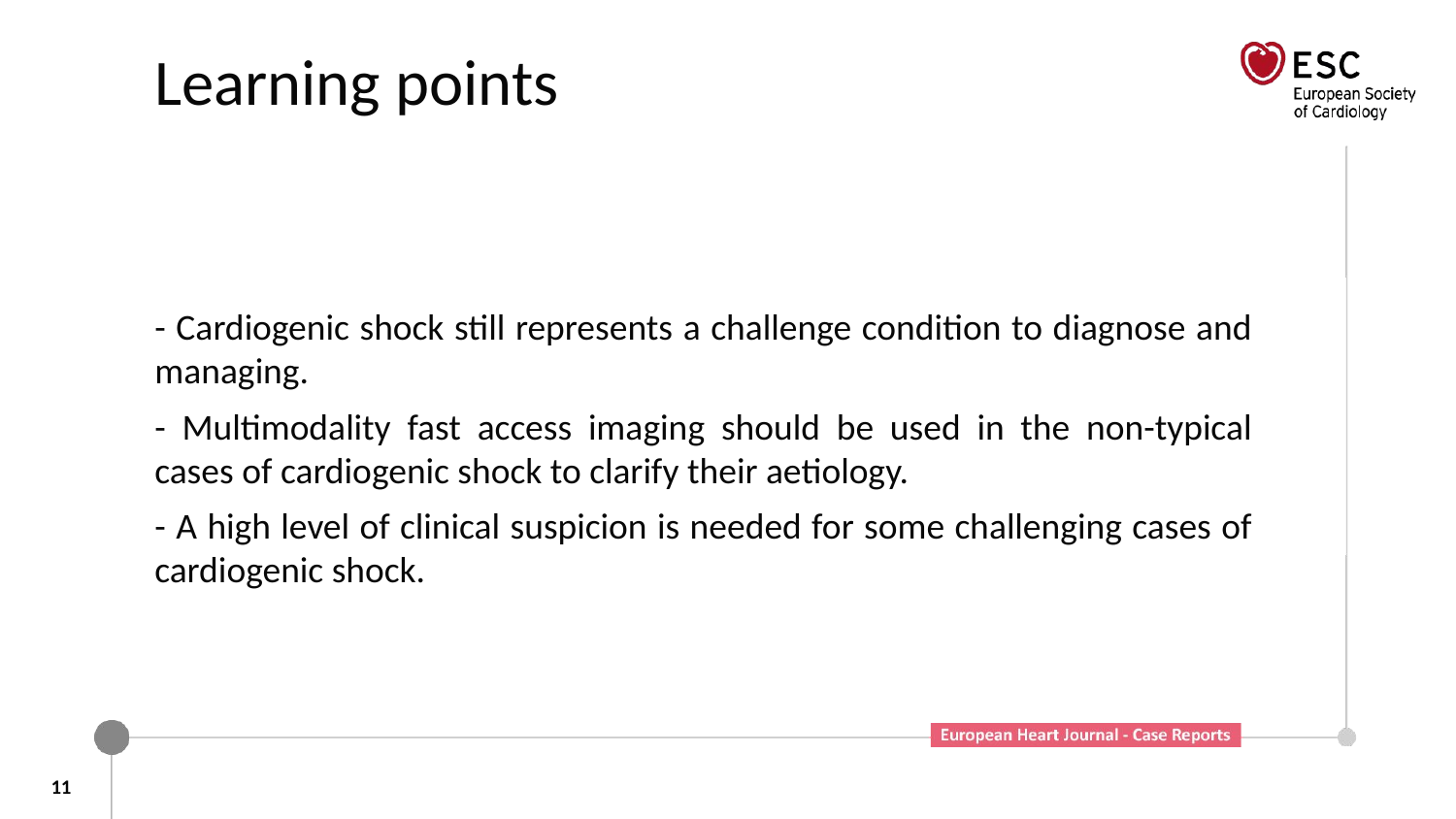

# Learning points
- Cardiogenic shock still represents a challenge condition to diagnose and managing.
- Multimodality fast access imaging should be used in the non-typical cases of cardiogenic shock to clarify their aetiology.
- A high level of clinical suspicion is needed for some challenging cases of cardiogenic shock.
11

## Slide 12
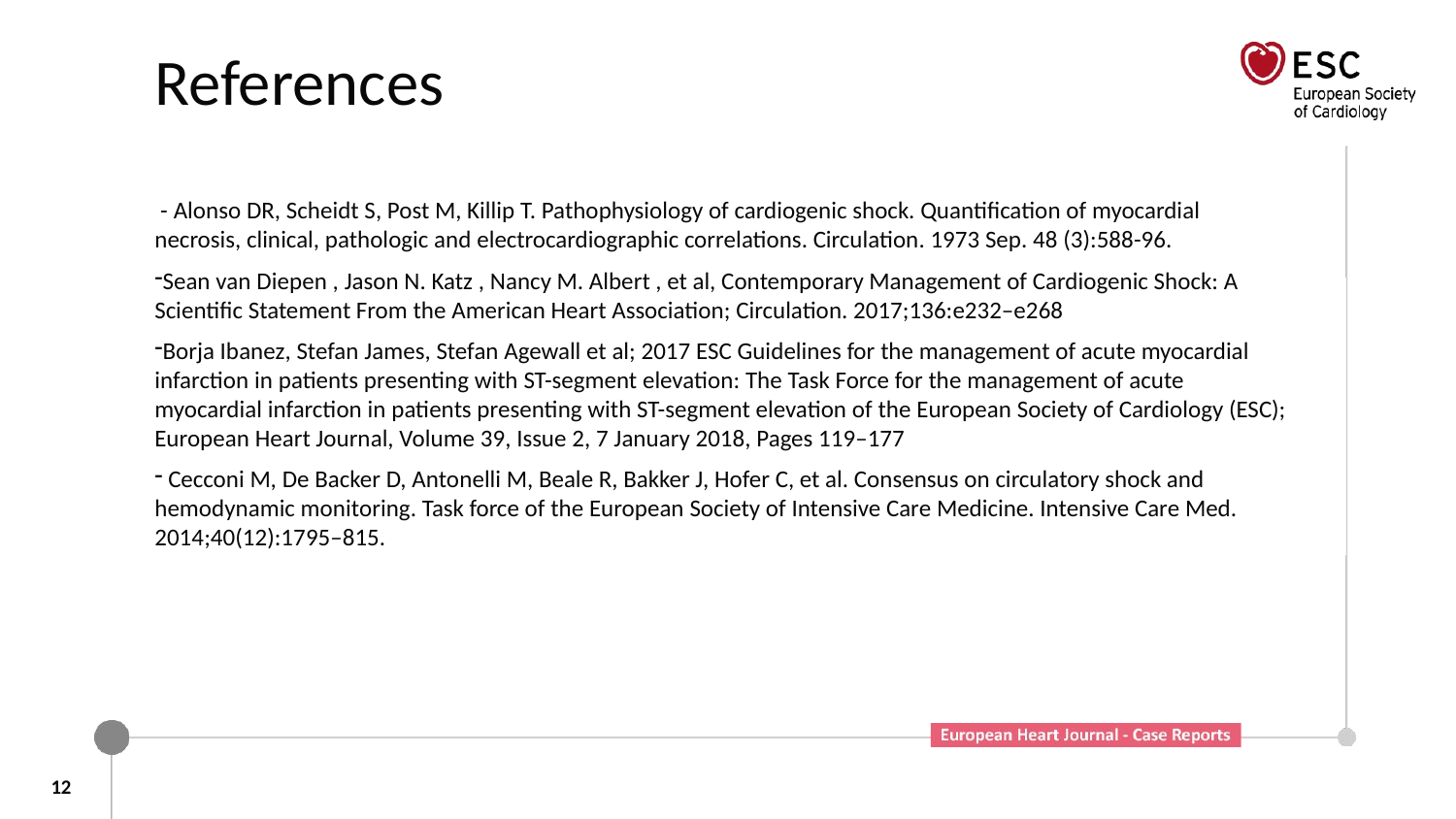

# References
 - Alonso DR, Scheidt S, Post M, Killip T. Pathophysiology of cardiogenic shock. Quantification of myocardial necrosis, clinical, pathologic and electrocardiographic correlations. Circulation. 1973 Sep. 48 (3):588-96.
Sean van Diepen , Jason N. Katz , Nancy M. Albert , et al, Contemporary Management of Cardiogenic Shock: A Scientific Statement From the American Heart Association; Circulation. 2017;136:e232–e268
Borja Ibanez, Stefan James, Stefan Agewall et al; 2017 ESC Guidelines for the management of acute myocardial infarction in patients presenting with ST-segment elevation: The Task Force for the management of acute myocardial infarction in patients presenting with ST-segment elevation of the European Society of Cardiology (ESC); European Heart Journal, Volume 39, Issue 2, 7 January 2018, Pages 119–177
 Cecconi M, De Backer D, Antonelli M, Beale R, Bakker J, Hofer C, et al. Consensus on circulatory shock and hemodynamic monitoring. Task force of the European Society of Intensive Care Medicine. Intensive Care Med. 2014;40(12):1795–815.
12
